# Rapid and Sensitive Detection of Polycyclic Aromatic Hydrocarbons in Tea Leaves Using Magnetic Approach

**DOI:** 10.3390/foods12112270

**Published:** 2023-06-05

**Authors:** Xiaohan Zhao, Xiao Feng, Jingwen Chen, Lanxin Zhang, Lingzi Zhai, Sizhe Lv, Yonghao Ye, Yongqi Chen, Tian Zhong, Xi Yu, Ying Xiao

**Affiliations:** 1 State Key Laboratory of Quality Research in Chinese Medicine, Macau University of Science and Technology, Taipa, Macao 999078, China; 2009853dct20003@student.must.edu.mo; 2 Key Laboratory of Grains and Oils Quality Control and Processing, Collaborative Innovation Center for Modern Grain Circulation and Safety, College of Food Science and Engineering, Nanjing University of Finance and Economics, Nanjing 210023, China; fengxiao@nufe.edu.cn; 3 Faculty of Medicine, Macau University of Science and Technology, Avenida Wai Long Taipa, Macao 999078, China; daisychan.must@gmail.com (J.C.); 1909853dh011003@student.must.edu.mo (L.Z.); 1909853eh011001@student.must.edu.mo (L.Z.); 2009853gh011005@student.must.edu.mo (S.L.); tzhong@must.edu.mo (T.Z.); 4 Zhuhai Resproly Pharmaceutical Technology Co., Ltd., Blk 11, International Health Port, No. 628, Airport West Road, Jinwan District, Zhuhai 519040, China; yonghaoye@resploy.com (Y.Y.); yqchen@resploy.com (Y.C.); 5 Guangdong-Hong Kong-Macau Joint Laboratory for Contaminants Exposure and Health, Guangzhou 510006, China

**Keywords:** polycyclic aromatic hydrocarbons, tea, magnetic solid phase extraction, UPLC, nanoparticles

## Abstract

A rapid and efficient method using an alkyl-functionalized magnetic nanoparticles-based extraction technique combined with Ultra-High Performance Liquid Chromatography was developed for the detection of trace amounts of polycyclic aromatic hydrocarbons in tea leaves. As a popular coating for chromatographic column packing materials, C_18_-alkyl has been demonstrated to be effective in separating polycyclic aromatic hydrocarbons. Additionally, the magnetism of the nanomaterials accelerates the extraction process while their high surface ratio enables desirable dispersity in the sample matrix. Meanwhile, the adsorbents can be washed and reused 30 times without compromising recovery, which greatly reduces the budget. The effects of various parameters were investigated and optimized, and the recoveries for five analytes were in the range of 84.8–105.4%. The RSD of intra-day and inter-day were below 11.9% and 6.8%, respectively. The limits of detection and limits of quantification ranged from 1.69–9.97 ng g^−1^ and 5.12–30.21 ng g^−1^, indicating satisfactory sensitivity. Thus, the proposed methodology is rapid, highly efficient, and economical, and it expands the application of magnetic cleanup approaches in complex food matrices.

## 1. Introduction

Tea is one of the most widely consumed beverages worldwide, and its status as the national drink of China has been recognized since ancient times. The global spread of Chinese tea leaves during the 16th century, facilitated by increased cultural exchange and trade, firmly cemented tea’s place as an important beverage. In particular, tea has had a profound impact on British culture, with many individuals consuming large quantities of it. Numerous scientific studies have identified potential health benefits associated with tea consumption, including cholesterol reduction, anti-microbial and hepatoprotective effects, and prevention of cardiovascular disease and cancer [1]. Residual chemical contaminants present in tea leaves due to air pollution deposition pose potential health hazards for consumers. Heavy metals and pesticide residues are among the primary pollutants that have undergone extensive investigation, and their detection and monitoring are crucial [2]. Based on the correlation between medicine and food, tea has been utilized for medicinal purposes in China for an extended period. Various active components present in tea exhibit antiviral properties; for example, some polyphenols have inhibitor potential by binding to the active site of SARS-CoV-2 RdRp, which means that it might serve as a precaution and treatment as a candidate drug for COVID-19 [3].

Polycyclic Aromatic Hydrocarbons (PAHs) are chemical structures composed of two to seven benzene rings, which can take a linear, angular, or agglomerated form, mainly due to the carbon-containing compounds, bioaccumulation in the food chain, and industrial food processing. At present, there are more than 100 known PAHs, while 16 PAHs are listed by the US Environmental Protection Agency as being regulated. The maximum residue limits (MRLs) of benzo(a)pyrene and four PAHs in certain foodstuffs, such as oils, fats, and smoked products, have been specified by the current EU regulation to be 2–5 μg kg^−1^ and 10–30 μg kg^−1^, respectively [4]. These compounds possess teratogenic, mutagenic, and carcinogenic properties, and their stable structure makes them slow to degrade [5]. The aromatic’s stable structure makes it hard for the PAHs to degrade. PAHs have been detected in a variety of food matrices, such as seafood, oil, barbecue, and so on [4,6]. While the content of PAHs is present in low amounts, research has shown a link between their presence and the development of various types of malignant tumors in laboratory animals and humans [7].

Most people drink tea without washing the tea leaves, which makes tea drinks a potential source of daily exposure to PAHs. Because of its long growth cycle and complex processing process, tea is susceptible to environmental pollutants. The main source of PAH contamination in tea stems from the absorption of PAHs from the atmosphere by tea leaves during their growing and drying stages [8]. When the electric heating drying mode replaces the conventional drying mode, PAHs in fresh leaves are one of the main sources of PAH contamination in tea due to the amplification effect during the processing [9]. Furthermore, tea plants are subject to PAH contamination during cultivation, primarily through the presence of these carcinogens in the air and soil. PAHs in the air may be adsorbed onto the surface of fresh tea leaves or directly absorbed into the tissues of the tea plants. Meanwhile, the roots of tea plants absorb PAHs present in the soil, which then migrate to the leaves [10]. Notably, evidence indicates that the contamination levels of PAHs in different teas differed. A survey conducted by Duedahl-Olesen (2015) showed that after detecting four PAHs in 18 tea leaves, the highest four PAH levels with a maximum of 115 ng g^−1^ were found in black tea leaves [11]. Li et al. (2011) reported that the mean PAHs of unfermented tea (green tea), semi-fermented tea (oolong tea), and fully fermented tea (black tea and Pu-erh tea) were 206.0 ng g^−1^, 153.0 ng g^−1^, and 202.8 ng g^−1^, which were obtained by rapid screening of three PAHs in a variety of tea samples [12]. Previous research had established that PAHs were detected in tea leaves sold in different regions. Adisa et al. (2015) analyzed and evaluated a total of 18 PAH congeners in an evaluation of 28 different dry tea samples sold in the U.S. The sum of the 18 PAH congeners, recorded as dry mass, ranged between 101 and 1337 ng g^−1^ [13]. Khiadani et al. (2013) showed that the average total PAHs in eight black teas commonly found in Iran ranged from 139 to 2082 ng g^−1^. Four rings of PAH compounds accounted for 46% of the total PAH compounds, they were the dominant compounds, and no PAHs with five or six rings were found [14]. Sadowska-Rociek et al. (2014) tested 22 different types of tea in Poland, detecting a total of 8 PAHs with a total content ranging from 30.1 to 147.1 ng g^−1^. Among them, white tea and green tea had a lower PAH content of 11.8 and 10.6 ng g^−1^, respectively, while black tea and red tea had a higher content of 16.9 and 17.0 ng g^−1^, respectively [15]. 

The determination of polycyclic aromatic hydrocarbons (PAHs) in matrices commonly involves sample extraction and preparation prior to chromatography analysis. The efficacy of the extraction methods is critical in developing high-accuracy analyses. To deal with the diversity and complexity of food matrices, many pretreatment techniques have been developed to separate and absorb PAHs, including solid-phase extraction (SPE), solid-phase microextraction (SPME), and stir-bar sorptive extraction (SBSE) [16,17]. However, there are several drawbacks to the methods: SPE is laborious, difficult, expensive, and not environmentally friendly; SPME is expensive, time-consuming, and has small sorption phase volumes, while method automation and memory effects in SBSE are restrictive. Furthermore, the low amounts of PAHs in complex samples make them difficult to detect using current methods. 

Recently, a proposed methodology combined with magnetic nanoparticles has been developed [18]. In many fields, magnetic solid-phase extraction (MSPE) has attracted increasing interest due to its simplicity, time-saving potential, and high accuracy in sample preparation [19]. Normally, magnetic nanoparticles (MNPs) are synthesized by grafting inorganic magnetic cores with organic functional groups [20]. Due to their excellent adsorption properties and stability, nano forms of the material are used in various research areas. J. Ding et al. (2010) reported the synthesis of magnetic material Fe_3_O_4_@3-(Trimethoxysilyl)propyl methacrylate@ionic liquid nanoparticles (Fe_3_O_4_@MPS@IL NPs) and applied as the adsorbent of seven heavy molecular weight PAHs from tea soup samples [21]. Additionally, magnetic nanoparticles coated with other substances, such as diphenyl functionalization Fe_3_O_4_ nanoparticles and carbon-ferromagnetic nanocomposites, have been utilized to absorb PAHs from different matrices [22]. 

However, the use of MSPE to determine trace analytes in solid samples is scarce. Additionally, there is a need to enhance the technique’s adaptation to complex matrix systems so that they can better assist highly demanding modern separation tasks. The aim of the proposed study is to extend the application of magnetic adsorbents in bulk and complex solid food samples, thereby allowing for simple, costless, and highly effective sample pretreatment. Alkyl groups with 18 carbons (C_18_), as a widely-used chromatographic material, are proven by many to be effective in the preconcentration of various organic contaminants due to their high adsorption capacity, outstanding stability, and strong separation power. Additionally, octadecylsilyl (ODS) is the most widely used reagent to graft C_18_ alkyl groups onto inorganic magnetic nanoparticles with the aid of a silica encapsulation layer in between [23,24]. This study aims to develop an extraction technique utilizing self-prepared C_18_-coated magnetic nanoparticles (C_18_/MNPs) to detect PAHs in tea samples. The use of MSPE in conjunction with UPLC results in a highly efficient preconcentration process for PAHs in tea, which is cost-effective, environmentally friendly, and highly accurate. The C_18_/MNPs are a valuable addition to the analytical tools available.

## 2. Materials and Methods

### 2.1. Real Sample

Tea leaves (Tianfeng, Fujian, China) (*Camellia sinensis* (*Camellia*, *C. sinensis*, *Theaceae*)) were purchased from the local supermarket in Macau in Oct 2021. The shelf life of the tea leaves is 540 days. The sample was first ground using a mortar and pestle before passing through a 24-mesh sieve. The portion of the sample that remained above the sieve’s cutoff was subjected to further grinding and sieving, and the process was repeated three times to ensure that the final particle size of the sample was below 0.85 μm. The as-prepared sample powder was then stored in a refrigerator to prevent moisture absorption prior to usage. 

### 2.2. Chemicals and Standards

PAHs standards of benzo[b]fluoranthene (99.4%), ben-zo[a]anthracene (99.0%), fluorene, anthracene (99.9%), and pyrene (98.5%), as shown in Table 1, were acquired from Manhage Bio-technology (Beijing, China). Analytical grade sodium hydroxide (NaOH), Iron (Ⅱ) chloride tetrahydrate, and Iron (III) chloride hexahydrate were obtained from Macklin. Tetraethyl orthosilicate (TEOS), isopropyl alcohol, ammonia, anhydrous toluene, triethylamine, chloro(diethyl)octadecylsilane (ODS, 95% *v*/*v*), hydrochloric acid (HCl, 37% *v/v*), and potassium bromide (KBr) came from Sigma Macau. Methanol (CH_4_O, polarity:6.6), 95% ethanol (C_2_H_6_O, polarity:4.3), acetonitrile (CH_3_CN, polarity:6.2), and acetic acid (CH_3_COOH, polarity:6.2) came from Macklin (Shanghai, China). Acidified acetonitrile (3% acetic acid, *v*/*v*) was prepared personally. Ultra-high performance liquid chromatography was from Shimadzu. HPLC-grade acetonitrile and methanol were provided by Anaqua (Cleveland, OH, USA).

### 2.3. Preparation and Characterization of C_18_/MNPs

#### 2.3.1. Synthesis of C_18_/MNPs

The C_18_/MNPs were prepared by chemical co-precipitation, salinization, and alkylation method [25].

**Figure 1:**
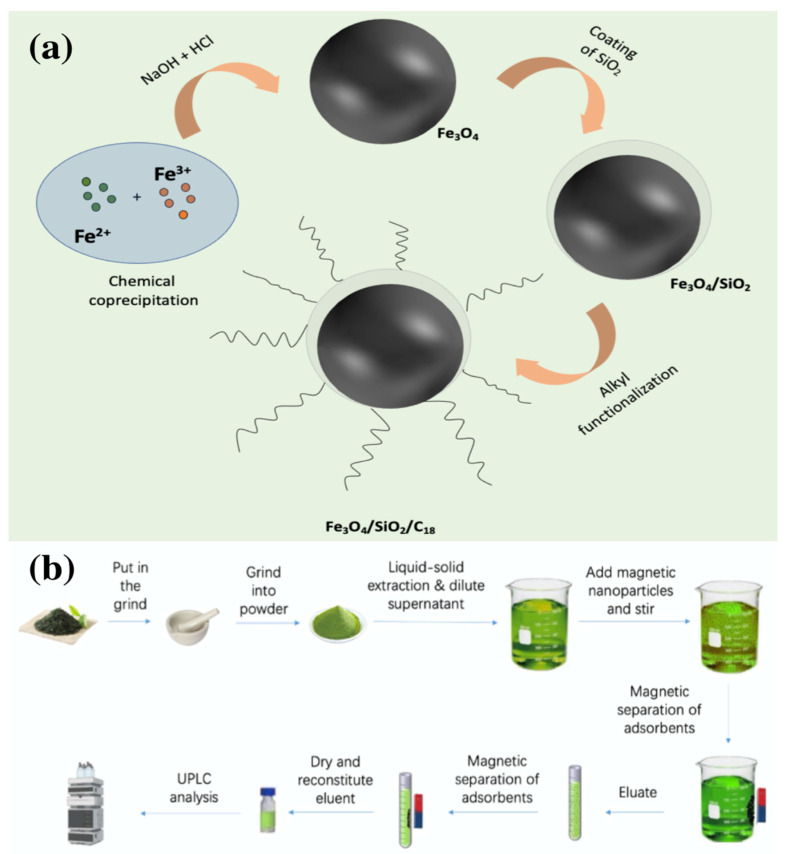
(**a**) Synthesis of C_18_/MNPs; (**b**) Protocol for the analysis of the tea powder using the proposed method.

Figure 1a shows that under the alkaline environment, using chemical co-precipitation to prepare the MNPs, FeCl_2_·4H_2_O (3.25 g) and FeCl_3_ (5.65 g) were added to DI water with HCl, respectively, followed by ultrasonication. The iron salt solution was added to NaOH solution (1.5 M) using a dropping funnel, stirred vigorously and heated to 80 °C in a water bath, and refluxed with nitrogen gas. The synthesis reaction was carried out for 2 h and washed three times with DI water. 

Then, the successfully synthesized MNPs were added to a mixture of DI water (6 mL), isopropanol (43 mL), and ammonia (25 wt%, 1.25 mL) and stirred for 15 min. TEOS (125 μL) was put into the solution with nitrogen gas. At room temperature, the solution was stirred for 4 h, washed thrice, and dried in a vacuum oven.

In short, the magnetic silica nanoparticles’ surface was covered on the surface by organic coatings through the alkyl. Dried magnetic silica microspheres (0.6 g) were added to anhydrous toluene (30 mL) and heated to boiling. Triethylamine (0.6 mL) and ODS (0.9 g) were added to the mixture and refluxed for 5 h. The successfully synthesized C_18_/MNPs were washed and dried.

#### 2.3.2. Characterization of C_18_/MNPs

The functional groups, hydrophobicity, roughness, particle size, and magnetic strength of the synthesized C_18_/MNPs, were analyzed by Fourier transform infrared spectroscopy (FT-IR), Brunaue–Emmett–Teller (BET), static contact angle (SCA), vibrating sample magnetometer (VSM), zeta-potential analysis, and the laser particle size analyzer. For FT-IR, small amounts of the nanoparticles were ground together with KBr at a 1:130 (*w/w*) ratio, and then they were pressed into flakes. The specific surface area and pore diameter were determined using Brunauer–Emmett–Teller surface area analyzer and a Micromeritics ASAP 2460 V3.01. Surface water contact angles of the scaffolds were measured using a contact angle meter (KRUSS DSA100) at in-room temperature, using a vibrating sample magnetometer (Lake Shore 7404) to collect the magnetization curves. For the size and surface zeta potential, the Nano-Particle size and Zeta Potential Analyzer (Malvern, Zetasizer nano ZS) were used. C_18_/MNPs were dispersed in deionized water, and zeta potential analysis and laser particle size analysis were performed after 30 min of ultrasound due to its easy magnetic aggregation.

### 2.4. C_18_/MNPs-Based Extraction Procedure for the Preconcentration and Detection of Five Kinds of PAHs in Tea

#### 2.4.1. Configuration of Standard Solution and Preparation of Spike Samples

Five kinds of PAHs were dissolved in acetonitrile to obtain spiked samples at concentrations of 300 ng mL^−1^. Spike samples (0.5, 1, 5, 10, 20, 30, 50, 100, 200, 300 ng g^−1^) were prepared by adding PAHs standard solution to the tea powder with a vortex to help mix. For example, a spiked sample at 10 ng g^−1^ was prepared by adding 500 μL of 300 ng mL^−1^ standard solution to 15 g of tea powder.

#### 2.4.2. Preconcentration Procedures

Figure 1b displays the protocol for the preconcentration of PAHs in tea samples with the aid of the self-prepared magnetic nanoparticles: First, acetonitrile (30 mL) was used to extract 15 g of tea powder with the aid of a 10 min vortex. After filtration, the supernatant was taken, and DI water (70 mL) was diluted. The C_18_/MNPs (110 mg) were added to adsorb PAHs, and the mixture vortexed for 30 min. The C_18_/MNPs absorbed the targeted analytes with a 3 × 15 mm (diameter/thickness) ultrapowerful Nd-Fe-B permanent magnet alloy (flux density, B = 200 roT). The acidified acetonitrile (5 mL) was added to the C_18_/MNPs and vortexed for 90 s. The C_18_/MNPs were separated and blown dry using a nitrogen-blowing apparatus at 35 °C (2 Nm^3^ h^−1^). Acetonitrile (200 μL) was used to reconstitute the residue for UPLC analysis.

### 2.5. Recycling of C_18_/MNPs

The used C_18_/MNPs were transferred into a beaker with the aid of methanol. After ultrasound treatment for 30 min, the MNPs were precipitated by an external strong permanent magnet. The C_18_/MNPs were washed thrice with methanol and water, dried overnight in an oven at 60.0 °C, and then stored in sealed centrifuge tubes for subsequent use.

### 2.6. UPLC Analysis

The UPLC analysis used a Luna 5u C_18_ column (150 mm length, 4.6 mm id, 100 Å pore size, Phenomenex, CA, USA) in a UPLC system equipped with a single-wavelength UV detector (Shimadzu, Japan). Analysis entailed maintaining the column oven at 30 °C, injecting 10 μL of the sample, and employing a flow rate of 0.3 mL min^−1^. The detection wavelength was set at 254 nm in order to achieve clear peaks for each PAH according to an existing method and our preliminary experiments [26]. Water and acetonitrile were employed as mobile phases A and B, respectively. The elution gradient for UPLC analysis was as follows: 0–15 min, 80% B; 15–40 min, 95% B; 40–45 min, 40% B; 45–50 min, 40% B. All the spiked, blank, and real sample extracts and standards were analyzed using the above UPLC method.

### 2.7. Statistical Analysis

To evaluate reliability between sample sets, three parallel optimization experiments and practical sample analyses were conducted, and sample data were analyzed using IBM SPSS Statistics. Under the optimization process, one-way analysis of variance (ANOVA) at *p* < 0.05 was performed for the individual sample to ensure the reliability of the triplicates.

## 3. Results and Discussion

### 3.1. Characterizing of C_18_/MNPs

**Figure 2:**
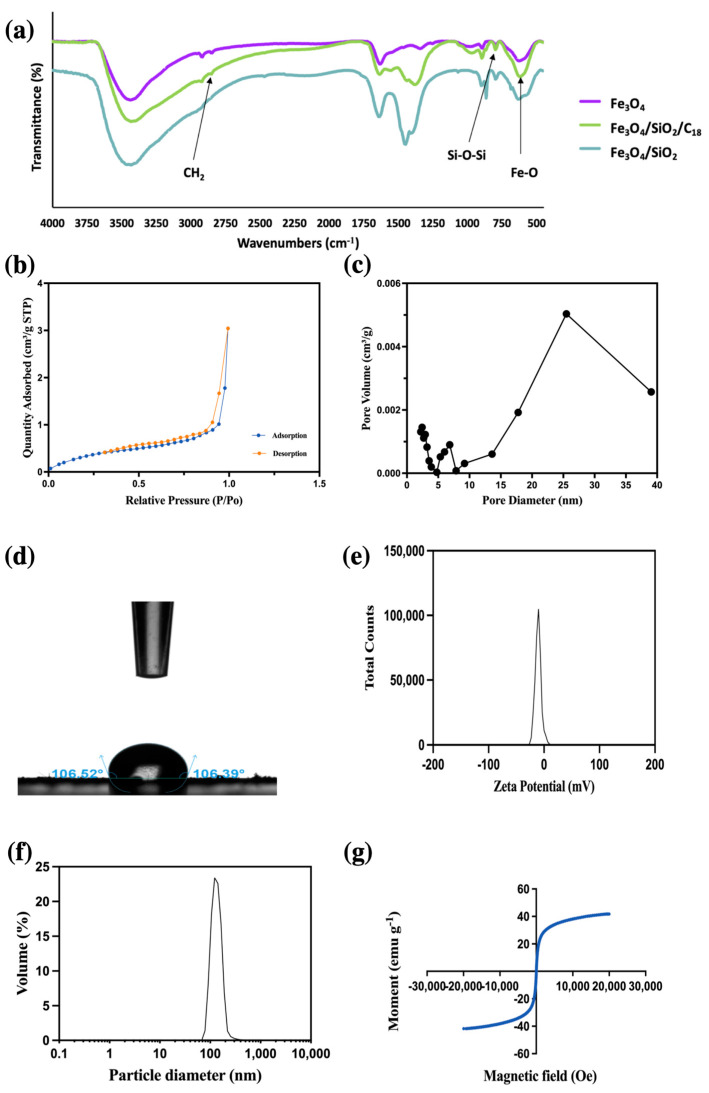
Characterizations of the magnetic C_18_/MNPs (**a**) Fourier transform infrared spectra; (**b**) Adsorption and desorption of nitrogen isotherms; (**c**) Pore distribution; (**d**) Static contact angle image; (**e**) Zeta-potential; (**f**) Particle size distribution; (**g**) Hysteresis loop.

The FT-IR spectra of the Fe_3_O_4_ nanoparticles, Fe_3_O_4_-SiO_2_ nanoparticles, and C_18_-coated magnetic nanoparticles are shown in Figure 2a. In the spectrum of the C_18_/MNPs, the absorption band around 590 cm^−1^ was caused by the stretching vibration of the Fe–O–Fe group, which indicated the existence of ferrite nanocores [27]. The presence of the peaks at around 1095 cm^−1^ ascertained the existence of the aromatic Si–O–Si group, verifying that the Fe_3_O_4_ nanoparticles were covered by a layer of SiO_2_ [28]. The absorption band around 2800–2900 cm^−1^ was indicative of the presence of the C_18_ layer due to the CH_2_ group originating from the silane coupling agent [28]. Therefore, it indicated that C_18_-coated ferro ferric oxide had been successfully synthesized. 

The adsorption capability of C_18_/MNPs was further characterized through nitrogen adsorption and desorption isotherms under 77 K. As given in Figure 2b, the N_2_ adsorption and desorption isotherms all displayed typical Type-IV, which was consistent with the 17.08 nm mesoporous structure of the C_18_/MNPs in Figure 2c. The calculated BET surface areas and the BJH cumulative pore volumes were 1.52 m^2^ g^−1^ and 0.004709 cm^3^ g^−1^, respectively [28]. 

The surface property of C_18_/MNPs was evaluated by static contact angles. The water contact angle of the as-prepared C_18_/MNPs shown in Figure 2d is estimated to be 106.5°, indicating that the C_18_/MNPs were hydrophobic [29].

The surface charge state of C_18_/MNPs were characterized by measuring the zeta potential of the particles in an aqueous solution. Figure 2e showed that the zeta potential of the C_18_/MNPs was −10.5 mV. The zeta potential value indicated that the physical stability was good [30].

The DLS analysis of the particle size distribution showed that the particle size of MNPs measured 104 nm, whereas the mean diameter of C_18_/MNPs was 143 nm. Such nano-level size distribution allows for good dispersal of the nanoparticles in the sample matrix and sufficient contact with the sample. Therefore, the nanoparticles are favorable to be used as adsorbents [31].

The magnetization curve obtained using the VSM in Figure 2g showed that the C_18_/MNPs were superparamagnetic since the magnetic curve displayed no hysteresis loop. The magnetization values were measured to be 53.2 emu g^−1^ for C_18_/MNPs. The result indicated that the magnetism of the C_18_/MNPs was high enough for the rapid magnetic separation from the sample matrix [32].

### 3.2. Optimizing MSPE Conditions 

The effects of various parameters were investigated and optimized to improve the extraction efficiency of MSPE [33]. All the optimization experiments were conducted in triplicates, and the averages were used for the bar charts.

#### 3.2.1. Amount of Absorbents

**Figure 3:**
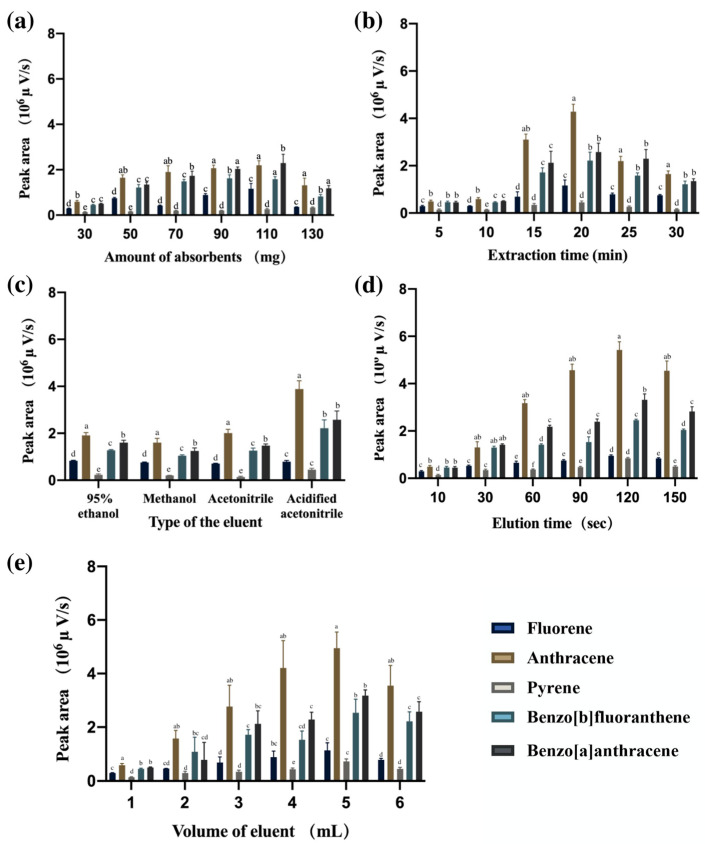
Optimizing MSPE conditions: (**a**) Amount of adsorbents; (**b**) Extraction time; (**c**) Type of the eluent; (**d**) Elution time; (**e**) Volume of eluent. Note: ND: Identical letters indicate insignificant differences, while different letters indicate significant differences (*p* = 0.05).

In Figure 3a, the extraction efficiency is positively correlated with the amount of adsorbent used. The increase in the adsorbent amount leads to an increase in the access sites of the adsorbent, thereby elevating the extraction efficiency [34]. According to the results of the statistical analysis, when the adsorbent content reached 90 mg, the peak height was not significantly different from that when the adsorbent content was 110 mg. However, an excessive increase in the adsorbent amount caused a decrease in the adsorption efficiency due to the limitation caused by specific sample volume and contact. Consequently, this can be accounted for in that 90 mg of C_18_/MNPs was chosen as the optimum use amount of adsorbents.

#### 3.2.2. Extraction Time 

Figure 3b shows that the stirring time had a significant influence on the extraction efficiency. In order to minimize the analysis time and maintain a high extraction efficiency within a shorter time frame, the extraction time was optimized within the range of 5–30 min. It was observed that after a stirring time of 20 min, no further increase could be achieved. This could be caused by the distribution equilibrium of the hydrophobic interaction between the PAHs and C_18_ groups on the magnetic nanoparticles [35]. Therefore, 20 min is set as the optimum extraction time.

#### 3.2.3. Type of the Eluent

Another major influencing factor was the type of elution solvent used. Solvents with different polarities might exert different elution power for certain analytes. This is because the intermolecular force is impacted by the square of the dipole moment of the molecule, which means two pair of molecules with similar dipole moment shares similar interaction power. Based on this, commonly used solvents with different polarities, including methanol (6.6), 95% ethanol (4.3), acetonitrile (6.2), and acidified acetonitrile (3% acetic acid, *v/v*), were compared for their effectiveness in desorbing PAHs. According to the results displayed in Figure 3c, acidified acetonitrile showed the highest elution power [35]. Therefore, acidified acetonitrile was settled as the elution solvent for the analysis.

#### 3.2.4. Elution Time

Figure 3d showed that 90 s was sufficient for the thorough elution of the PAHs. This can be attributed to the equilibrium time needed for the complete mass transfer of the analyte from the adsorbent to the organic solvent [34]. 

#### 3.2.5. Volume of Eluent

Next, the volume of eluent had a certain effect. The desorption solvent volume effect was investigated by varying the solution volume from 1 mL to 6 mL. As can be seen from Figure 3e, the peak areas of PAHs increased with the increase in desorption solvent volume and reached a plateau at 5 mL. The optimal solvent volume should be able to eluate the analytes from the adsorbents within a relatively small consumption of the solvent. Therefore, 5 mL was set as the optimum volume [36]. 

### 3.3. Evaluation of the Detection Method Performance 

**Figure 4:**
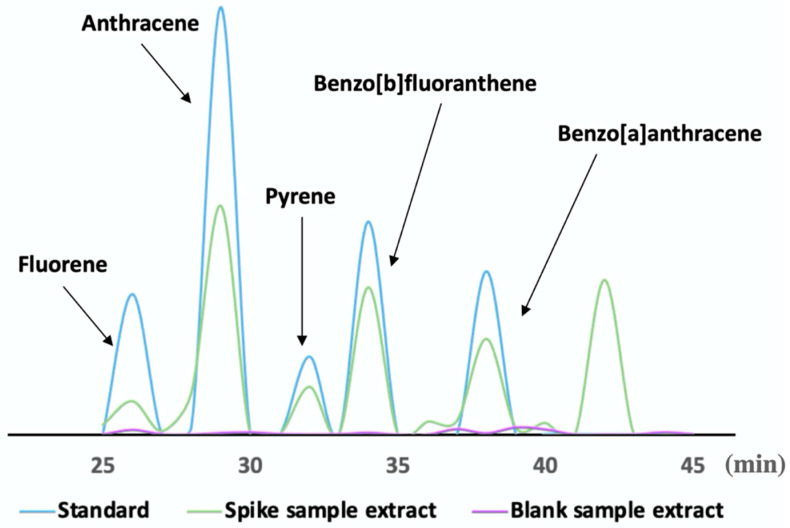
UPLC chromatograms of the standard solution of 5 PAHs, the spiked sample extract, and the blank extract.

The proposed methodology helped the acquisition of PAH calibration curves for different concentrations of tea samples (0.5, 1, 5, 10, 20, 30, 50, 100, 200, and 300 ng g^−1^). Figure 4 shows PAHs mixed standard solution (300 ng mL^−1^), spiked sample extract (300 ng g^−1^), and blank sample extract of the chromatographic results. The calibration curves of five PAHs were obtained using the optimal extraction conditions. Table 2 summarized that the R^2^ of all calibration curves was greater than 0.997 within the linear range of the analytes, reflecting the result that the method was accurate enough to be suitable for quantitative purposes. The method was further proof of the feasibility of the magnetic nanoparticle enrichment of PAHs in tea samples, which had proven to be more sensitive than traditional methods. When the limits of detection (LODs) and limits of quantification (LOQs) were determined at their lowest concentrations is the point at which the five PAHs can be detected at signal-to-noise ratios above 3 and 10. To draw the proposed method’s accurate LOD and LOQ values, each analyte sample took ten times analysis.

The LOD and LOQ values for the five PAHs of interest were determined in the range of 1.69–9.97 and 5.12–30.21 ng g^−1^, indicating that the demonstrated method had good sensitivity, which is consistent with other studies that used magnetic nanoparticles [18,21].

### 3.4. Reproducibility and Reusability of the C_18_/MNPs

To validate the precision of the C_18_/MNPs as adsorbents for PAHs in tea, the optimized parameters were used to test the reproducibility of the method that was applied within intra-day and inter-day. To test the reproducibility of the proposed method, the same experiment was repeated three times in one day and on three separate days under the optimized conditions. The samples of tea powder were spiked at 50, 100, and 200 ng g^−1^, respectively. The result is shown in Table 3. It suggests that the intra-day recovery for PAHs falls within the 81.1–102.1% range, with RSD ranging from 1.3% to 11.9%. Similarly, the inter-day experiment showed recovery between 84.8% and 105.4%, with RSD ranging from 2.3% to 6.8%. The intra-day RSD values were compromised compared with that of inter-day. 

**Figure 5:**
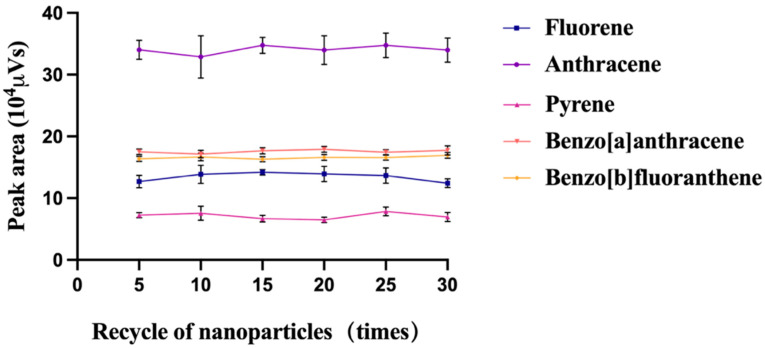
Effect of recycling times on the peak areas of PAHs.

After repeating the experiment, the relationship between the UPLC signal of the spiked tea powder extract and the reuse time was displayed in Figure 5. The results suggested that the extraction effectiveness of nanoparticles had no significant change during the repeating extraction procedure, indicating the nanoparticles had good stability and durability. Peak areas of PAHs had RSDs by 8.4% for Fluorene, 5.8% for Anthracene, 10.8% for Pyrene, 3.0% for Benzo[a]anthracene and 2.7% for Benzo[b]fluoranthene, respectively. Thus, it was proved that the recyclability of C_18_/MNPs was great. Compared to those traditional methods using disposable adsorbents, recyclable magnetic nanoparticles can reduce environmental pollution and costs [37]. 

### 3.5. Method Comparison

In Table 4, a comprehensive evaluation of the proposed methodology was carried out by comparing it with other methods. The main factors were listed, including linear range, LOD, and recovery, and the advantages and disadvantages of each method were also analyzed. While traditional methods enable the analysis and detection of PAHs, we found that we require a significant amount of time and a large volume of solvent and are likely to cause secondary pollution. The MSPE detection method has a wider linear range, relatively low RSD, and is capable of preserving the environment by utilizing fewer organic solvents and reusable adsorbents. We used UPLC-UV was utilized in lieu of high-precision instruments such as mass spectrometers, given its commonality, easy accessibility, and low budget. At the same time, the MSPE method can also adapt to different detection needs by changing the selective enrichment material, which has strong flexibility and applicability.

### 3.6. Real Sample Analysis

Thirteen different brands of tea samples were procured from the Macau supermarket chain between 2020 and 2022. For each brand, three packages were purchased from three different shops. The tea samples were stored at room temperature prior to analysis, and the analysis was conducted at least a month prior to their expiration date.

Commission Regulation (EU) No. 2015/1933 of 27 October 2015 listed already-dried herbs, for which the maximum allowed BaP content may not exceed 10 µg kg^−1^ and total 4PAHs (Benzo(a)pyrene, Benzo(a)anthracene, Benzo(b)fluoranthene, Chrysen) 50 µg kg^−1^. However, the MRLs for PAHs in tea were not specified. Here, we used the maximum allowable content for PAHs in herbs to assess the exposure risk of PAHs in tea. Only Benzo(a)anthracene (BaA) and Benzo[b]fluoranthene (BbF) are currently regulated among target PAHs of this experiment and are described with high toxic equivalent factors (TEF). The test results of real samples are shown in Table 5. The content of BaA and BbF was far below the limit or not detected in both black and green tea. A previous study using ultrasonic extraction to detect PAHs in dried black and green tea found high levels of Fluorene (Flu) (mean value 95.2 ng g^−1^ and 134.9 ng g^−1^, respectively) and relatively low levels of Benzo(a)anthracene (BaA) (mean value 20.9 ng g^−1^ and 28.4 ng g^−1^, respectively). In addition, a certain amount of BbF was also detected (mean value 9 ng g^−1^ in both dried black and green tea) [38]. This study is mostly consistent with previous results. We found a high content of Flu (mean value 132.3 ng g^−1^ in green tea, 109.8 ng g^−1^ in black tea) and BaA with a maximum content of 8.4 ng g^−1^ in green tea; such differences may be caused by the environmental pollution in the tea-growing region and tea processing techniques. Furthermore, we also found a relatively high amount of Anthracene (Ant) and Pyrene (Pyr), which has not been reported by other research; since there are no regulations about PAHs in dried tea, we strongly advise that the health risks of Pyr can be estimated and we hope that our study can provide theoretical support to the establishment of the standards.

In addition, many studies have reported the highest total amount of ∑16PAH in dry black tea. Black tea is made by fully fermenting tea leaves for several hours, followed by smoking or steaming. Combustion of firewood produces PAHs, so black tea is expected to absorb PAHs through the smoke [43]. Green tea, on the other hand, is made from fresh young leaves without fermentation. However, the essential oils in the tea can act as co-solvents for many lipophilic substances, thereby increasing the solubility of PAH in tea infusion [44]. The high temperature during black tea manufacturing may reduce the content of essential oils in the tea [45]. These studies, including this study, indicated the low health risk of PAH exposure associated with tea consumption [46].

## 4. Conclusions

In general, an MSPE platform was developed as a methodological innovation to the current pretreatment approaches for solid food matrices. Currently, the MSPE techniques are mainly confined to liquid matrices, which has hindered its expansion. The proposed methodology improved its application, enabling the pretreatment of a wider variety of samples. In this experiment, self-prepared C_18_-functionalized ultrafine magnetic silica nanoparticles were used for the detection of PAHs in tea with the extraction efficiency parameters optimized. Under the optimum conditions, the method was assayed and yielded satisfactory linearity, sensitivity, and reproducibility. The fine particle size of the nanoparticles contributed to their high specific surface area and adsorption sites. Additionally, C_18_/MNPs can be isolated by using permanent magnets after extraction, which means a simple and fast pre-processing method for instrumental analysis. The ability to reuse the nanoparticles considerably reduces costs and pollution, making this approach promising for solid food analysis, including barbecue, coffee, and similar samples. Overall, the developed method of MSPE with magnetic solid phase extraction is expected to serve as a promising platform for monitoring food safety.

## Figures and Tables

**Table 1:** The chemical structures composed of five PAHs.

Chemical Name	Abbreviation	Molecular Structure	Molecular Formula	Molecular Weight	Ring	Type
Fluorene	FLU	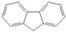	C_13_H_10_	166.22	3	Light PAHs
Anthracene	ANT	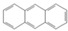	C_14_H_10_	178.23	3	Light PAHs
Pyrene	PYR	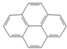	C_16_H_10_	202.25	4	Light PAHs
Benzo[a]anthracene	BaA	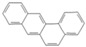	C_18_H_12_	228.3	4	Light PAHs
Benzo[b]fluoranthene	BbF	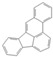	C_20_H_12_	252.3	5	Heavy PAHs

**Table 2:** Linearity, limits of detection (LODs), and limits of quantification (LOQs) of the proposed method for analyzing PAHs in tea.

Analyte	Equation of Calibration Curve	R^2^	LOD (ng g^−1^)	LOQ (ng g^−1^)
Fluorene	y = 48627x + 71642	0.998	7.93	24.03
Anthracene	y = 106034x + 4318855	0.999	5.32	16.12
Pyrene	y = 22396x + 216848	0.997	9.97	30.21
Benzo[a]anthracene	y = 56517x + 357848	0.998	1.69	5.12
Benzo[b]fluoranthene	y = 51622x + 661315	0.998	5.43	16.45

**Table 3:** Inter-day and intra-day reproducibility of 5 PAHs.

Analyte	Concentration (ng g^−1^)	Intra-Day Precision		Inter-Day Precision	
		Recovery ± SD (%)	RSD (%)	Recovery ± SD (%)	RSD (%)
Fluorene	50	94.8 ± 2.8	3.4	84.8 ± 2.9	6.8
	100	95.2 ± 0.7	1.3	91.6 ± 0.3	3.1
	200	88.9 ± 1.9	1.4	93.6 ± 6.1	6.6
Anthracene	50	91.7 ± 0.8	2.9	88.1 ± 0.9	5.9
	100	100.2 ± 1.6	1.3	94.6 ± 0.7	3.7
	200	93.0 ± 0.2	6.7	99.0 ± 2.3	2.3
Pyrene	50	89.9 ± 1.2	5.9	99.3 ± 0.7	2.8
	100	95.8 ± 1.4	2.3	100.5 ± 1.7	4.7
	200	102.1 ± 0.7	11.9	105.4 ± 3.0	2.9
Benzo[a] anthracene	50	99.1 ± 1.1	2.1	93.2 ± 1.8	5.1
	100	97.4 ± 0.9	2.4	96.7 ± 0.6	6.2
	200	81.1 ± 2.2	2.2	86.3 ± 3.6	4.2
Benzo[b] fluoranthene	50	102.1 ± 3.1	7.1	96.2 ± 2.1	4.9
	100	98.2 ± 1.7	2.9	98.5 ± 5.2	5.2
	200	93.4 ± 3.5	5.2	91.4 ± 3.6	4.0

**Table 4:** Comparison between the proposed and other methods for the analysis of PAHs in tea/coffee.

Method	Detection Technique	Sample	Linear Range (ng g^−1^)	LOD (ng g^−1^)	Recovery (%)	RSD (%)	References
SPE	GC-MS	Dry tea	-	0.09–0.32	37.00–96.10	-	Ciemniak et al. (2019) [38]
SPE	HPLC-FLD	Tea	-	16.00	90.00–95.00	<10.00	Stuppner et al. (2020) [39]
LLE	GC-MS	Roasted coffee	0.25–4.00	0.04–0.18	87.08–111.28	3.26–23.75	Pissinatti et al. (2015) [40]
GE	UPLC-Q-TOF-MS	Tea	-	2.00–100.00	78.40–109.20	1.33–9.72	Li et al. (2022) [41]
UAE	GC-FID	Dry tea	-	0.30	90.24–108.92	77.02–100.60	Benson et al. (2018) [42]
MSPE	UPLC-UV	Tea	0.50–300.00	1.69–9.97	84.80–105.40	2.30–6.80	This work

Note: LLE, GE, and UAE stand for Liquid-liquid Extraction, Gradient Elution, and Ultrasonic Assisted Extraction, respectively. GC-MS, HPLC-FLD, UPLC-Q-TOF-MS, GC-FID, and UPLC-UV stand for Gas Chromatography-Mass Spectrometer, High-Performance Liquid Chromatography-Fluorescence Detector, Ultraperformance Liquid Chromatography–Quadrupole Time-Of-Flight Mass Spectrometry, Gas Chromatography-Flame Ionization Detector, and Ultra-Performance Liquid Chromatography-Ultraviolet, respectively.

**Table 5:** Real sample analysis of tea.

Tea Type		Fluorene (ng g^−1^)	Anthracene (ng g^−1^)	Pyrene (ng g^−1^)	Benzo[a]anthracene (ng g^−1^)	Benzo[b]fluoranthene (ng g^−1^)
Green (10 tea brands)	Mean	132.3	41.9	109.5	N.D.	N.D.
	Min	31.2	N.D.	N.D.	N.D.	N.D.
	Max	N.D.	196.5	N.D.	8.4	N.D.
Black (3 tea brands)	Mean	109.8	21.7	94.6	N.D.	N.D.
	Min	81.9	N.D.	70.3	N.D.	N.D.
	Max	141.5	31.2	113.4	5.3	N.D.

Note: N.D.: not detected or beyond the linear range.

## Data Availability

The data used to support the findings of this study can be made available by the corresponding author upon request.
